# A meta-analysis of the effects of cognitive behavioral therapy on quality of life and negative emotions of informal cancer caregivers

**DOI:** 10.3389/fpsyt.2022.979158

**Published:** 2022-11-23

**Authors:** Shuang Zhou, Yumei Wang, Qiang Wang, Guodong Yang, Huipeng Ren, Yanping Bao

**Affiliations:** ^1^Mental Health Center, Hebei Medical University and Hebei Technical Innovation Center for Mental Health Assessment and Intervention, Shijiazhuang, Hebei, China; ^2^Hebei Clinical Research Center for Mental Disorders and Institute of Mental Health, Shijiazhuang, Hebei, China; ^3^Department of Psychiatry, The First Hospital of Hebei Medical University, Shijiazhuang, Hebei, China; ^4^National Institute on Drug Dependence and Beijing Key Laboratory of Drug Dependence, Peking University, Beijing, China

**Keywords:** anxiety, cancer, cognitive behavioral therapy (CBT), depression, informal caregivers (IC), quality of life

## Abstract

**Objective:**

This study aimed to systematically review the effect of cognitive behavioral therapy (CBT) in terms of improving the quality of life (QOL) and negative emotion of informal cancer caregivers.

**Methods:**

PubMed, Cochrane Library, EMBASE, Web of Science, MEDLINE, China National Knowledge Infrastructure (CNKI), and China Biology Medicine disc (CBMdisc) were searched from the database establishment to October 2021. Literature screening, data extraction, and quality evaluation were conducted based on inclusion and exclusion criteria. Stata 14.0 software was used for data analysis.

**Results:**

A total of 17 randomized controlled trials (RCTs) involving 2,348 cancer informal caregivers (CGs) were included in this study, with an overall loss rate of 13.3%. Meta-analysis showed no statistically significant difference in the impact of CBT on QOL (*SMD* = 0.28, 95%CI: −0.09–0.65, *P* < 0.001). However, the improvement of depression in CBT intervention group was significantly better than that in control group (*SMD* = −0.32, 95%CI: −0.56 to −0.07, *P* = 0.010). The HADS subgroup showed differences in depression scores (*SMD* = −0.80, 95%CI: −1.30 to −0.29, *P* = 0.002). The overall effect of CBT on anxiety was statistically different, the improvement of anxiety in CBT intervention group was significantly better than that in control group (*SMD* = −0.36, 95%CI: −0.720–0.004, *P* = 0.047).

**Conclusions:**

CBT had a positive effect on reducing depression and anxiety in informal cancer caregivers, and the effect on quality of life was not statistically significant, but showed a positive trend.

**Systematic Review Registration:**

https://inplasy.com/generate-invoice/, identifier: INPLASY202230120.

## Introduction

With the incidence of cancer increasing year by year (1). Not only do cancer patients experience great pain during long-term treatment, but their caregivers have significant cognitive, emotional, and behavioral impacts. Caregivers are divided into formal and informal caregivers. The former refers to remunerative individuals, such as doctors, nurses, assistants, etc., while the informal caregivers involved in this study refer to the latter, namely unpaid individuals, usually referring to family members, spouses, neighbors or friends, etc., (2). An estimated 10–60% of Informal caregivers experience negative psychological and physiological sequelae, including anxiety (3), depression (4, 5) and physical disorders (6). It is increasingly recognized that comprehensive care for patients with cancer involves attending to the psychosocial needs of their informal caregivers (7). The current Intervention studies on caregivers can be divided into three types: education and information support, psychosocial support, and a combination of the two (8). Education and information support programs aim to provide skills related to symptom management or problem solving, while psychosocial support programs typically include counseling, face-to-face interviews, or cognitive behavioral interventions that can be delivered to an individual, spouse, or group over the phone, the Internet, or face to face. Among these interventions, cognitive behavioral therapy (CBT) has attracted the maximum attention. CBT refers to the general term of a treatment method that corrects wrong cognition, eliminates bad emotions and negative behaviors by changing people's thinking, belief and behavior (9). It covers a variety of psychological treatment methods, changes unreasonable cognitive concepts through correction technology, and closely links cognitive correction with behavior correction (10, 11). Create a virtuous circle between the two to replace the original vicious circle, so that the negative symptoms are alleviated and disappear (12). Its application in other psychosomatic fields has been proved to effectively reduce mental stress and negative emotions (8), and to enhance disease-related knowledge and improve awareness among participants. Existing evidence from randomized controlled trials suggests that CBT and various modified CBT are effective in improving negative emotions in caregivers of cancer patients, but there is a lack of evidence from systematic reviews of the efficacy of CBT in caregivers of cancer patients (13). Therefore, the aim of this study was to systematically evaluate the effects of cognitive behavioral therapy on informal cancer caregivers.

Considering the progress in studies concerning cancer caregivers since 2011, psychosocial interventions to caregivers are not just confined to one single outcome variable; instead, more than two outcome variables are taken into consideration. For example, some studies involved interventions directed on the quality of life (QOL), depression, anxiety, sleep, or self-efficacy of caregivers (14). A large number of randomized control groups no longer adopt a no-intervention policy when it comes to dealing with. All these factors make system review difficult. Therefore, this study only selected QOL, depression and anxiety scores reported in most randomized controlled trials as outcome indicators, and selected the control group as routine mental health education or related studies without intervention for meta-analysis.

## Methods

### Search strategy

Publications from establishment of the database to October 2021 were systematically selected. A literature search was conducted in the following digital databases: PubMed, the Cochrane Library, EMBASE, Web of Science, MEDLINE, China National Knowledge Infrastructure (CNKI), and China Biology Medicine DISC (CBMdisc). The search strategy followed the PICO model: Population: informal cancer caregivers; Intervention: cognitive behavioral therapy; Comparison: routine nursing/health education, or blank control; Outcome: QOL, depression, or anxiety.

Keywords related to oncology (cancer OR neoplasm OR oncology OR palliative care OR palliative medicine OR malignancy) were combined with keywords related to the population (caregiver OR carer OR caregiving OR spouse OR relative OR partner OR family) and the intervention (CBT OR iCBT OR cognitive therapy OR behavioral intervention OR cognitive intervention OR coping skills OR psychosocial OR problem-solving OR cognitive restructuring OR exposure OR mindfulness OR meditation OR relaxation training OR cognitive behavior therapy OR cognitive behavioral therapy OR psychotherapy). In addition, a backward search (snowballing) of reference lists of identified studies was conducted, and earlier systematic reviews together with a forward search (citation tracking) until no additional relevant studies were found.

### Selection strategy

All studies selected for final inclusion met the following criteria: (1) interventions must include informal caregivers, alone or with patients with cancer. (2) The intervention content of the study should conform to the CBT content standard, include at least one of the following components, and be considered as CBT (15): cognitive recombination, imaginary or in-body exposure, coping skills training, problem solving, behavioral activation, structured work, reception-based cognitive intervention, and managing stress through relaxation or mindfulness. (3) Participants were randomly assigned to either the intervention group or the control group of the study. The randomized controlled trial (RCT) method. (4) Psychosocial health indicators including QOL, depression, or anxiety were included. (5) The studies were published in Chinese or English.

In addition, studies involving children with cancer were excluded as well as those involving drug interventions, because of the nature of the parent-child relationship. All previous studies were evaluated independently by two researchers, and disagreements over the inclusion/exclusion of the study were resolved by consensus. A literature search focused only on studies published in peer-reviewed journals to enhance the rigor of the methodology examined.

### Review strategy

A pair of raters reviewed relevant studies and extracted data. In consideration of the heterogeneity of interventions and data, this study adopted the Cochrane Reviewer Handbook 5.1.0 (16) as the risk-of-bias (ROB) assessment tool to evaluate the overall quality of the study: random allocation method, allocation concealment, blinding (investigator-blinded and/or participant-blinded), integrity of result data, selective reporting of research results, and other sources of bias. All studies were scored as possessing (a) low risk of bias, (b) unclear, or (c) high risk of bias. Disagreements between researchers were resolved through discussion with a third reviewer.

### Data extraction

Two researchers conducted data extraction by reading the full text to determine the indicators for analysis independently. Descriptive data were extracted based on the following four aspects: literature characteristics, participant characteristics, intervention plan, measurement indicators, and test tools. Literature characteristics included author and year of publication. Participant characteristics included types of cancer, number of participants, and average age. The intervention plan included intervention content and follow-up. Measurement indicators and test tools included testing tools for QOL, depression, and anxiety in patients with cancer. The outcome indicators included QOL, depression, and anxiety measurement results (see Table 1 for specific measurement scale).

**Table 1:** Study characteristics.

**Source**	***N*** **(Int/Con)**	**Relation to patients**	**Age** **(M/SD)**	**Cancer type**	**Intervention**	**Control group**	**Follow-up**	**Measurement tools**
Bultz et al. (17)	34 (17/17)	Spouse	51	Breast	CBT 6 weeks	No intervention	3 months	POMS,IM SFSSS
Kuijer et al. (18)	59 (32/27)	Spouse	Nil	Breast, intestinal, Hodgkin, brain, lung	CBT	No intervention	3 months	CES-D
Hudson et al. (19)	106 (54/52)	Spouse, parent, child	60.78 (13.98)	Unspecified/ advanced	Psycho educational intervention:	Standard care	Nil	HADS
Given et al. (20)	237 (118/119)	Spouse and others	55.3 (13.76)	Unspecified/67% advanced	CBT	No intervention	10 weeks	CES-D
Carter et al. (21)	34 (16/18)	Spouse and children	53 (17)	Unspecified/advanced	Caregiver sleep intervention	No intervention	2\3\4 months	PSQI,CES-D CQOL-C
Badger et al. (22)	71 (36/35)	Partner	61.13 (10.9)	Prostate	Dyadic telephone counseling	Routine health education	8\16 weeks	CES-D PANAS,MFI
Meyers et al. (23)	476 (348/128)	Spouse, children, and others	61.4	Gastrointestinal, genitourinary, and thoracic	Paired home care guide	No intervention	30\90\120 \180\days	CHO-QOL SPSI-R
Fegg et al. (24)	133 (81/79)	Spouse, parent, and others	54.3 (13.2)	Gynecologic al tumor, lung, breast, and Brain tumor	Existential behavioral therapy	No intervention	3\12 months	BSI,EF WHOQOL-BR
Han et al. (25)	309 (154/155)	Spouse, children, and sibling	Nil	Unspecified	CBT	No intervention	Nil	SF-36 CQOL-C
Clark et al. (26)	131 (65/66)	Nil	58.7 (10.6)	Gastrointestinal, brain, head, neck, lung, and other	Paired structured CBT training	No intervention	4\27 weeks	CQOL-C
Dionne-Odom et al. (27)	124 (63/61)	Nil	60	Palliative care	Telephone grief counseling	No intervention	3 months	CQOL-C CES-D
Borji et al. (28)	80 (40/40)	Spouse, adult, other	Nil	Prostate	CBT	No intervention	8 weeks	DASS-21
Wu et al. (29)	60 (30/30)	Nil	Nil	Terminal stage	Psychological suggestion	No intervention	Nil	HAMD HAMA
Yue-cai et al. (30)	120 (60/60)	Nil	57.2	Oropharyngeal	CBT	General care	Nil	WHOQOL-100
Kubo et al. (31)	31 (17/14)	Spouse, child, other relatives, friend	Nil	Breast, hematologic, skin, lung, gastrointestinal, head, neck, genitourinary, and other	Mindfulness-based program	No intervention	Nil	HADS CQOL-C
Xiu et al. (32)	157 (81/76)	Family members	53.9 (12.18)	Lung	CBT	I-BMS	8\12 weeks	CQOL-C HADS, ISI
Trevino et al. (33)	26 (12/14)	Spouse, other	64.24 (16.74)	Lung, gynecologic, breast, colorectal, lymphoma, genitourinary, and pancreatic.	Managing anxiety from cancer	No intervention	Nil	HADS caregiver quality of life–cancer scale

Con, control group; Int, intervention group; POMS, the profile of mood states; IMS, the index of marital satisfaction; FSSS, the DUKE-UNC functional social support scale; CES-D, the center of epidemiological studies depression scale; HADS, the hospital anxiety and depression scale; PSQI, the Pittsburgh sleep quality index; CQOL-C, the caregiver quality of life index-cancer; PANAS, the positive and negative affect schedule; MFI, the 20-item multidimensional fatigue inventory; COH-QOL, the city of hope quality of life; SPSI-R, social problem-solving inventory-revised; BSI, the brief symptom inventory; WHOQOL, World Health Organization quality of life measurement scale; SF-36, MOS 36-item short-form health survey; DASS-21, the 21-item depression anxiety stress scales; HAMD, Hamilton depression scale; HAMA, Hamilton anxiety rating scale; ISI, 7-item insomnia severity index (see Appendix for a brief description of the scale).

### Data analysis

To accurately extract the data, a researcher extracted the data, and a second researcher confirmed the extracted data to ensure accuracy. Using Stata 14.0 software for meta-analysis, we adopted the random-effects model because different measuring tools were used to measure the same outcome. For continuous data, the standardized mean difference (SMD) was selected as the effect scale index for statistics. The magnitude of effect indicated the degree of influence of CBT on informal cancer caregivers. The effect values were all expressed in a 95% confidence interval (CI). Heterogeneity was explored using *Q* and *I*^2^ statistics. *Q*-tests were related to the probability that the results reflected systematic between-study differences. A *P* ≤ 0.10 was used to determine significant heterogeneity because of the generally low statistical power of heterogeneity tests. The *I*^2^ statistic was an estimate of the degree of observed heterogeneity unexplained by sampling error and was unaffected by the number of studies. *I*^2^ values of 0, 25, 50, and 75% were considered negligible, low, moderate, and high, respectively. Subgroup analysis, meta-regression and sensitivity analysis were conducted to explore the source of heterogeneity. Funnel plots and Egger's test were used to assess the presence of any publication biases.

## Results

### Literature search

**Figure 1:**
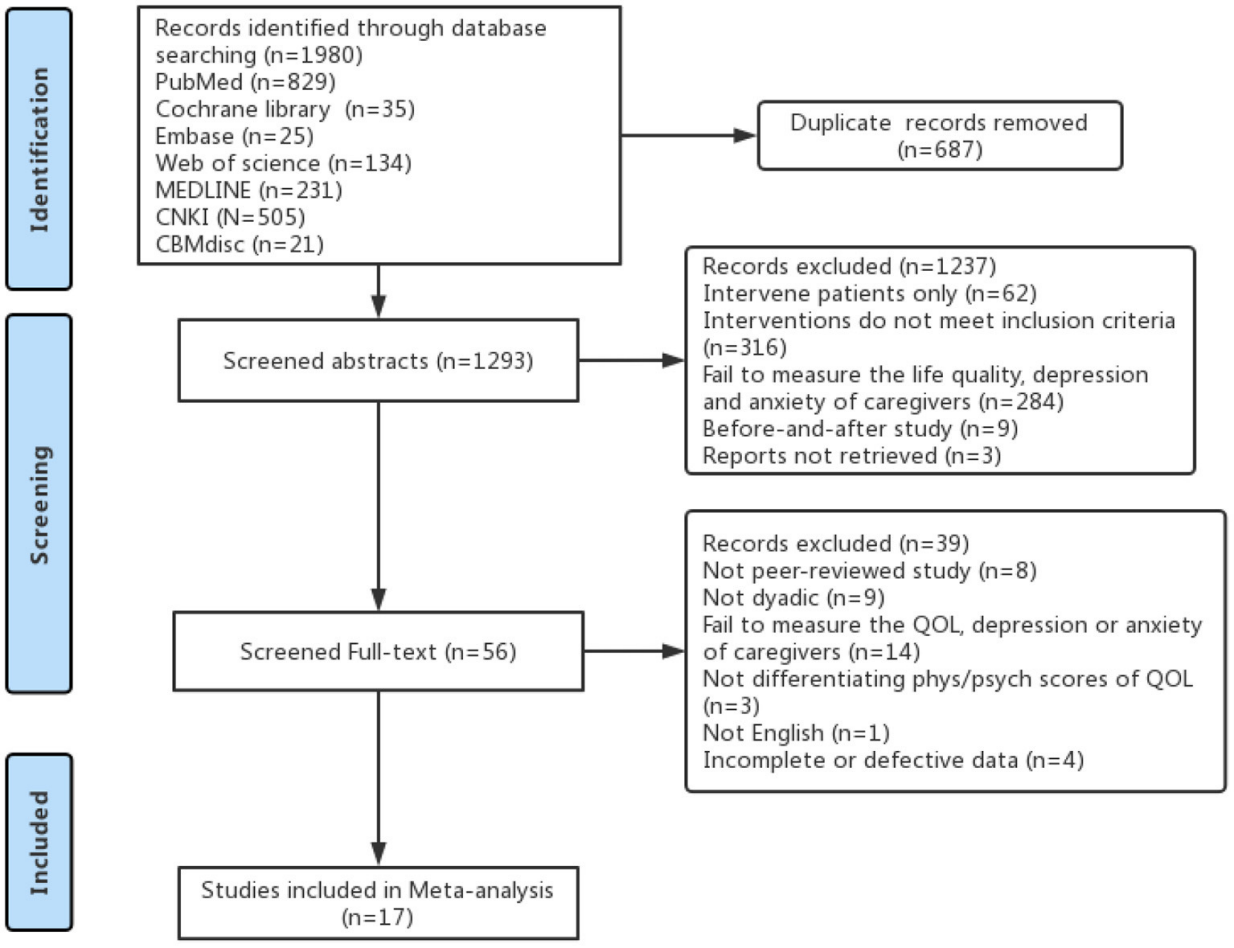
PRISMA flow diagram of the study selection procedure.

First, the researcher preliminarily screened 1980 relative studies in the literature. After removing duplicates, screening titles and abstracts, and reviewing the full text, 17 studies met the criteria, with 2,348 informal cancer caregivers. All of 17 studies reported the changes in life quality, depression, or anxiety of informal cancer caregivers after the intervention. A summary of the results of the literature search and screening process is shown in Figure 1.

### Study characteristics

A summary of all study characteristics is shown in Table 1. A total of 17 RCTs meeting the inclusion criteria on CBT treatment of informal cancer caregivers published between 2000 and 2021 were included, with 2,348 cancer caregivers enrolled at baseline and the number of caregivers in each study ranging from 26 to 476. The CBT in the intervention group were based on different theories, mainly including experience support, equality theory, structured intervention, problem solving and counseling, skill learning, mindfulness, meditation, stress management, etc. The Intervention duration ranged from 4 weeks to 3 months, and specific intervention nodes could not be counted. In the control group, four studies (19, 22, 30, 32) described general psychological support and none of the others had specific interventions, among them, the control group of five studies (18, 20, 21, 27, 31) was set as the waiting or delayed intervention group. Among all studies, the overall participant attrition rate was 13.3% and the study attrition rate varied from 0 to 57.5%. The reasons for attrition included the worsening or death of patients and the business of cancer caregivers.

The average age of the caregivers was 57.25 and were primarily spouses, adult children, parents, siblings, friends or significant others of cancer patients. Except for studies that did not report cancer types, almost every study included spouses/partner. Three studies specifically focused on spouses or partners (17, 18, 22). In terms of cancer types, one studies specifically focused on patients with breast cancer (17), two studies were specifically designed for prostate cancer (22, 28), one on patients with oropharyngeal carcinoma (30), and one on patients with lung cancer (32). Furthermore, one study focused on a specific cancer stage of patients: terminal stage (29). while another study focused only on patients who received palliative care (27). The rest of the study consisted of patients with different types of cancer.

### Risk of bias

**Figure 2:**
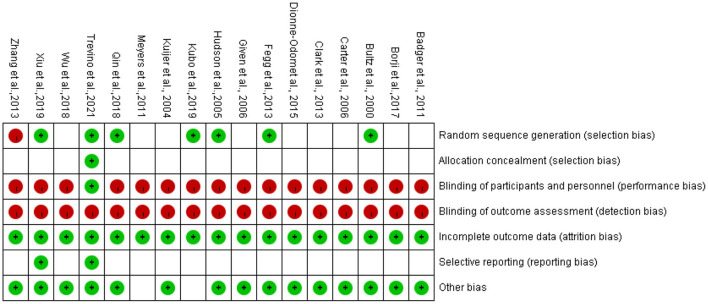
Risk of bias in individual studies. +, low risk of bias; –, high risk of bias; the blank represents uncertain risk.

The results of the ROB assessment are shown in Figure 2. Eight studies reported the generation of randomized controlled sequences, including simple randomization of stratified module grouping, drawing from the envelope, and computer-generated randomization listing and grouping according to the time of admission (high risk of bias), while the others were not described. Only one study reported allocation concealment and blinded participants; no studies blinded the evaluation of results. The data of all studies were relatively complete, although their selective publication was unclear. One study (23) might have had bias due to the unbalanced baseline data. Another study (31) might have increased the risk of bias in the results because two researchers did not independently coded the post-intervention interview transcript.

### Data synthesis

#### Effects of CBT on the QOL

**Figure 3:**
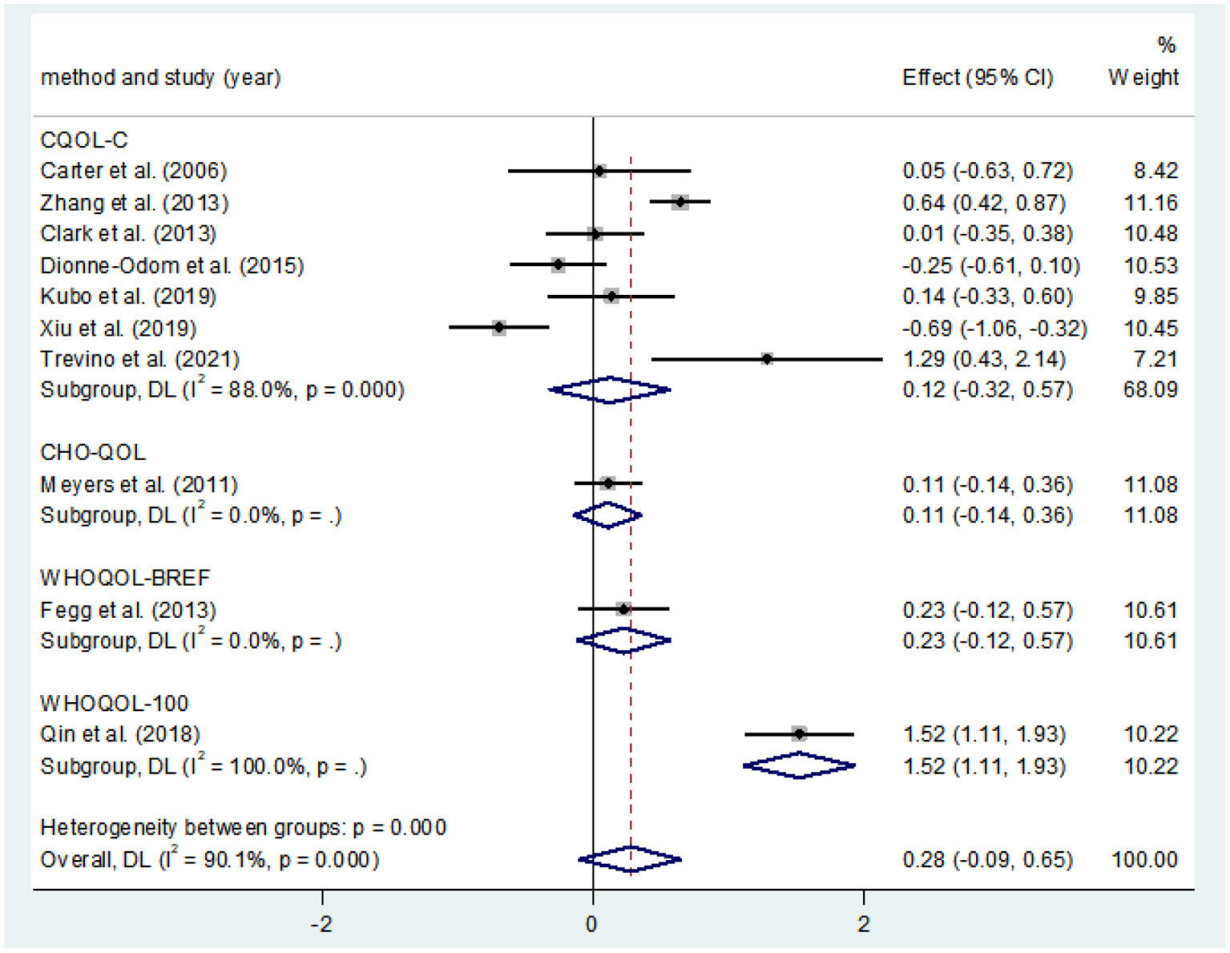
Forest maps for subgroup analysis of effects on QOL.

**Figure 4:**
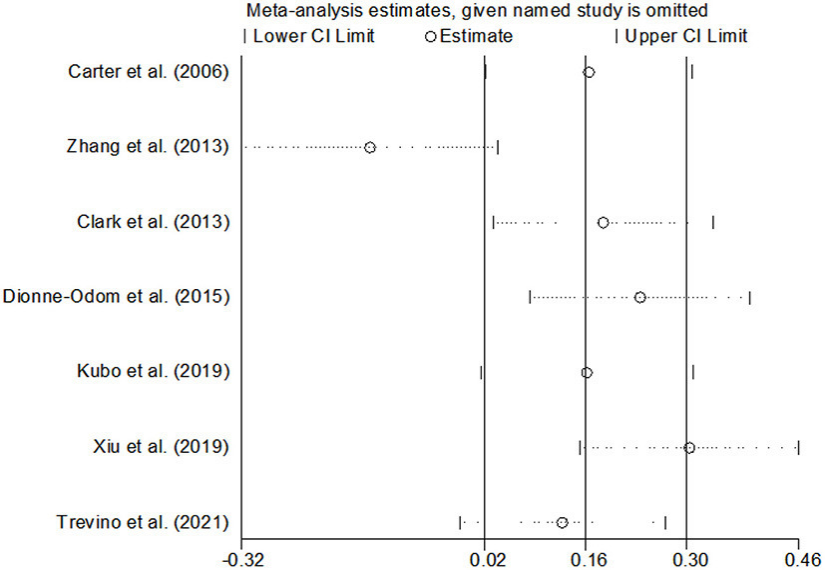
Sensitivity analysis of CQOL-C subgroup.

**Figure 5:**
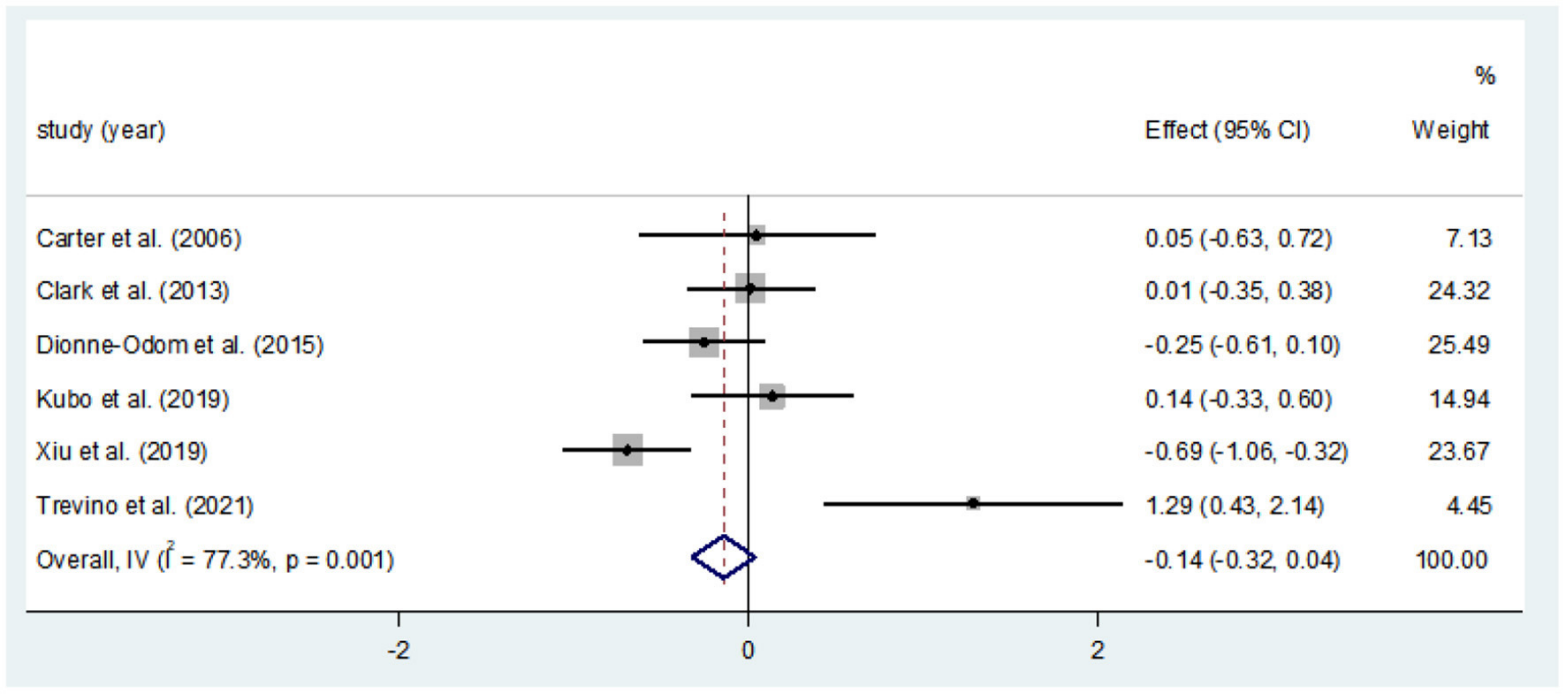
Forest map for estimating the effect size of QOL in CQOL-C subgroup [excluding Han et al. (25)].

**Figure 6:**
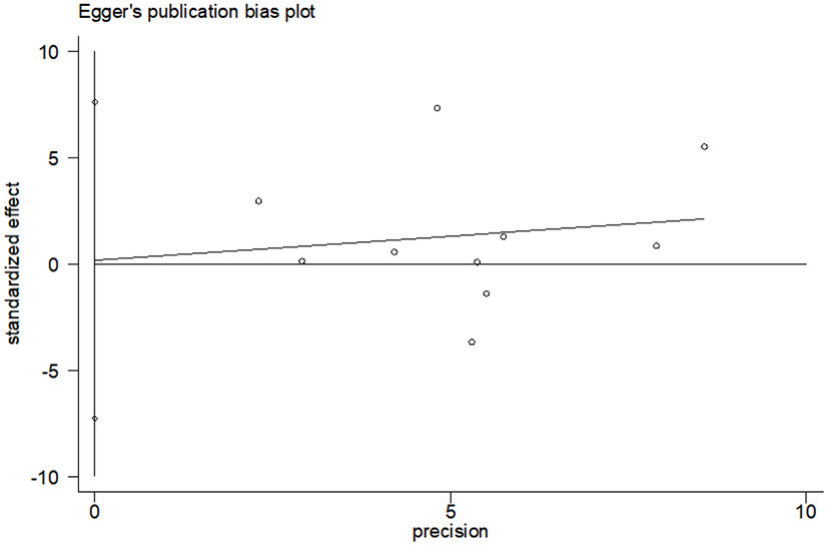
Publication bias test of CBT effect on QOL.

Ten studies compared the two groups with QOL. Seven studies adopted CQOL-C, while the other three were different. Subgroup analysis was performed according to different measuring tools, and the results showed that there was no significant difference in quality of life between the two groups (*SMD* = 0.28, 95%CI: −0.09–0.65, *P* < 0.001), the forest plot of pooled effect estimate for quality of life in Figure 3. There was high heterogeneity in CQOL-C subgroup (*SMD* = 0.12, 95%CI: −0.32–0.57, *P* = 0.029; *I*^2^ = 88.0%, *P* < 0.001), meta-regression analysis showed that publication age was not correlated with inter-study heterogeneity (*P* = 0.958). The results of sensitivity analysis are shown in Figure 4. When studies with higher risk of bias were excluded (25), the heterogeneity of the CQOL-C subgroup was reduced (*I*^2^= 77.3%, *P* = 0.001), but the difference in QOL score between the two groups was still not statistically significant (*SMD* = −0.124, 95%CI: −0.32–0.04, *P* = 0.117), and the forest map of effect estimation is shown in Figure 5. Egger's test results are shown in Figure 6, without significant publication bias (*P* = 0.960).

#### Effects of CBT on depression

**Figure 7:**
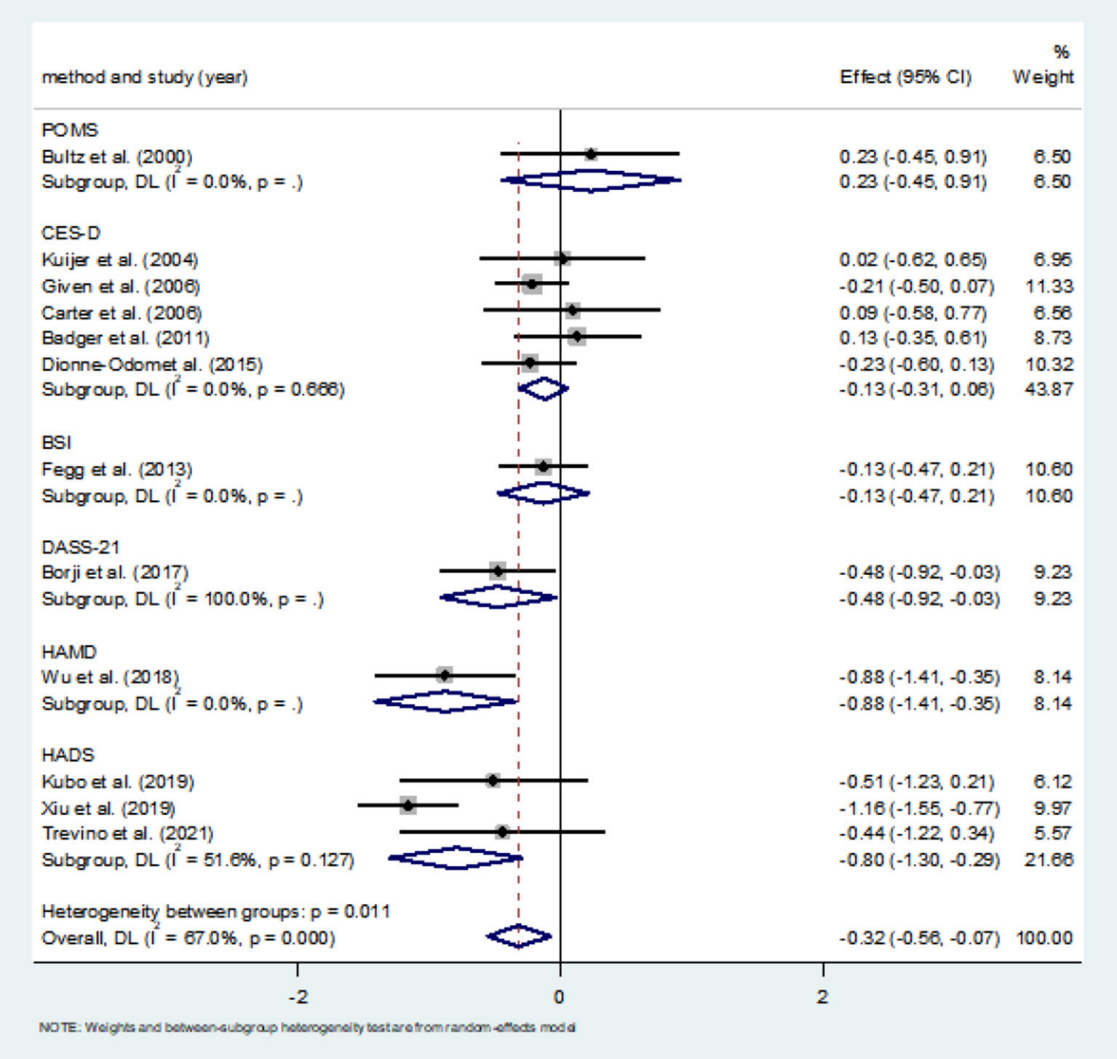
Forest maps for subgroup analysis of effects on depression.

**Figure 8:**
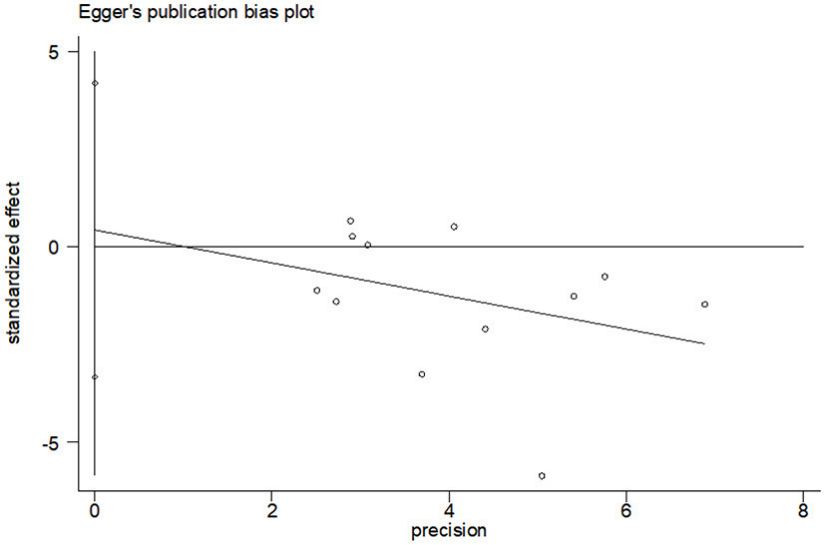
Publication bias test of CBT effect on depression.

A total of 12 studies compared depression scores between the two groups. Among them, CES-D was used in five studies to measure depression score, and HADS was used in three studies [Hudson et al. (19) used HADS, but only anxiety score was reported], and the other four studies used different measurement scales. Meta-analysis showed that the depression score of the CBT group was significantly lower than that of the control group (*SMD* = −0.32, 95%CI: −0.56 to −0.07, *P* = 0.010), with moderate heterogeneity (*I*^2^ = 67.0%, *P* < 0.001), and the forest map estimated by comprehensive effect is shown in Figure 7. Subgroup analysis showed significant difference in depression scores in HADS subgroups (*SMD* = −0.80, 95%CI: −1.30 to 0.29, *P* = 0.002; *I*^2^ = 51.6%, *P* = 0.127); the heterogeneity was almost negligible in CES-D subgroups (*I*^2^ = 0.0%, *P* = 0.666), but there was no significant difference in depression scores (*SMD* = −0.80, 95%CI: −1.30 to −0.29, *P* = 0.002). There was no significant publication bias (*P* = 0.803), and the Egger's test results were shown in Figure 8.

#### Effects of CBT on anxiety

**Figure 9:**
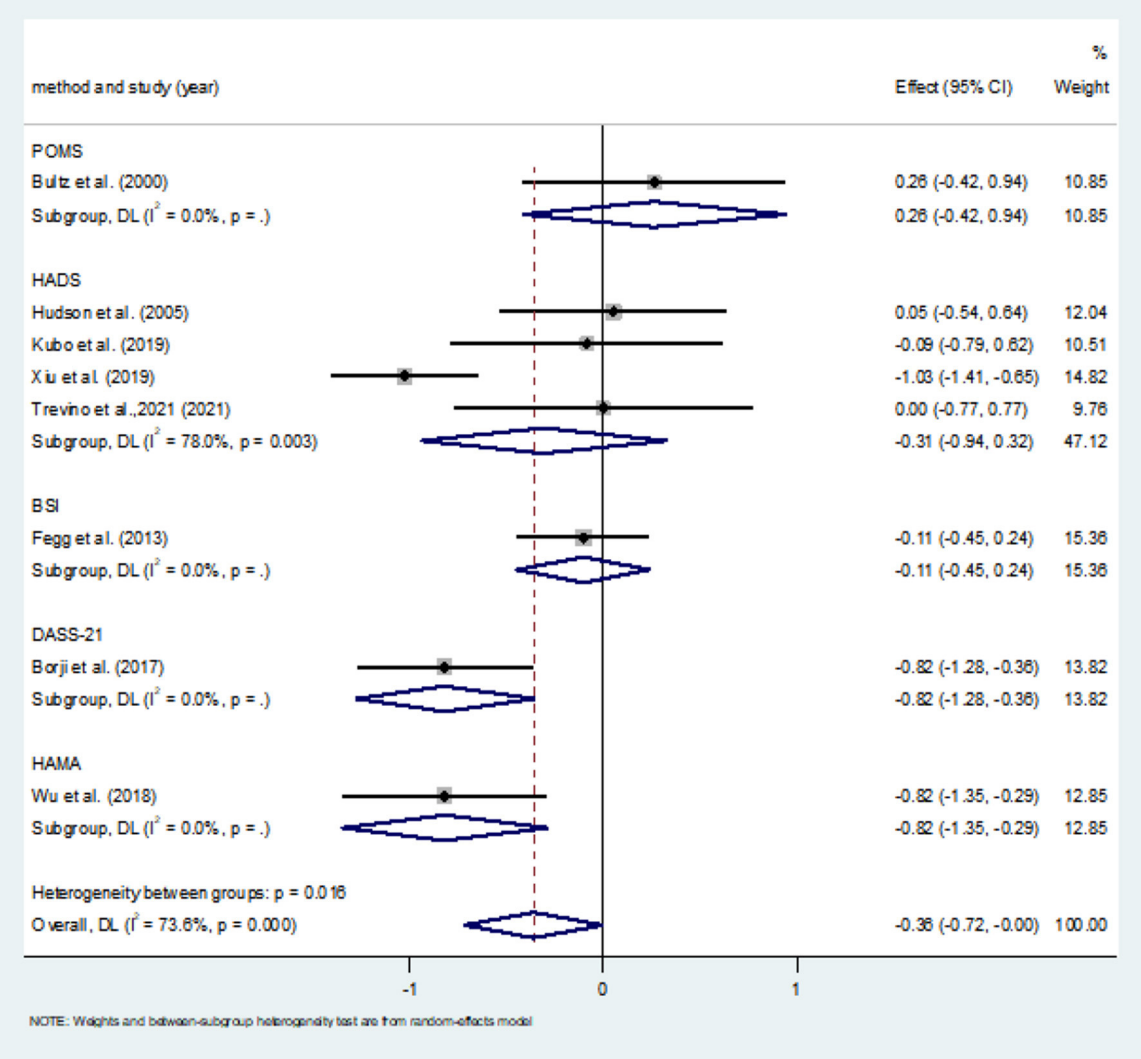
Forest maps for subgroup analysis of effects on anxiety.

**Figure 10:**
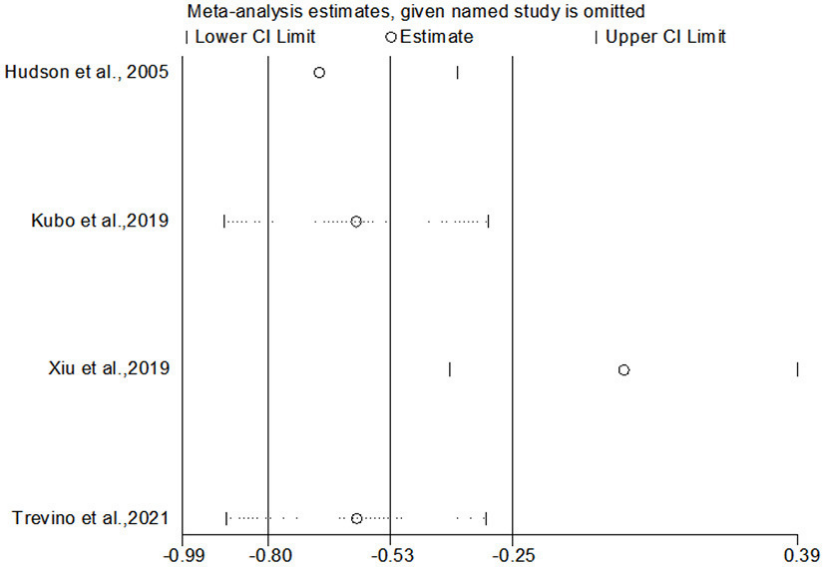
Sensitivity analysis of HADS subgroup.

**Figure 11:**
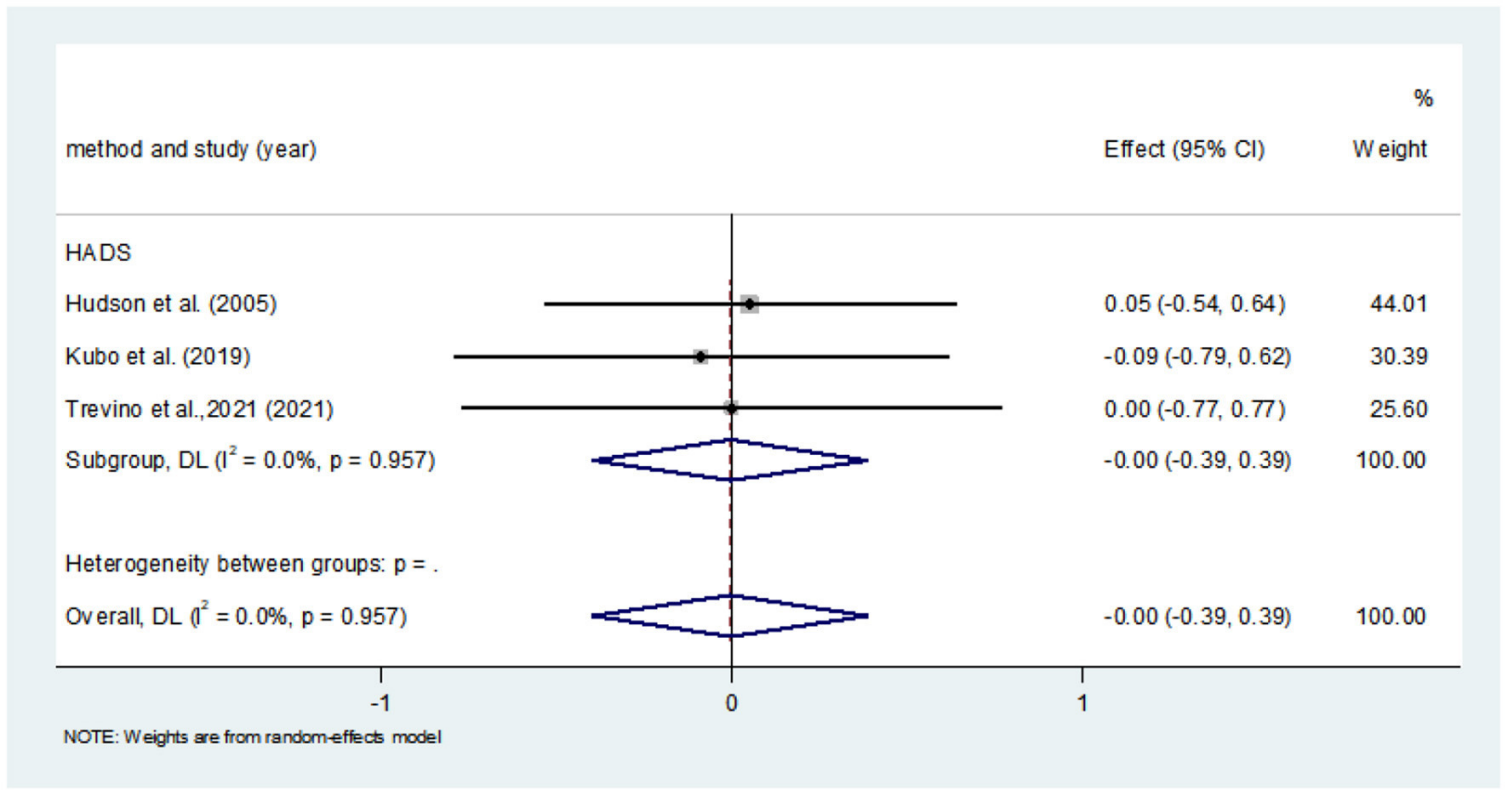
Forest map for estimating the effect size of anxiety in CQOL-C subgroup [excluding Xiu et al. (32)].

**Figure 12:**
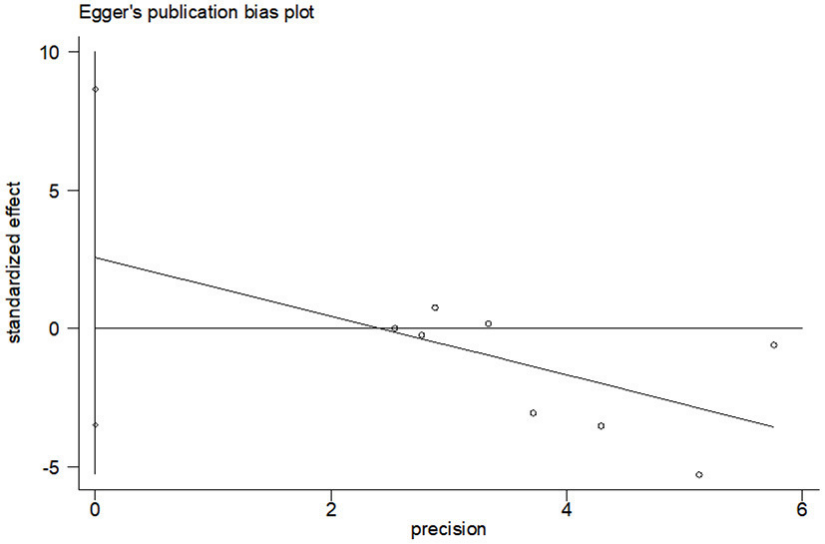
Publication bias test of CBT effect on anxiety.

Eight studies compared anxiety scores between the two groups after the intervention. Four of the studies used HADS to measure anxiety scores, and the other four used different scales. Meta-analysis showed statistically significant difference in anxiety scores between the two groups (*SMD* = −0.36, 95%CI: −0.720 to −0.004, *P* = 0.047), with moderate heterogeneity (*I*^2^ = 73.6%, *P* < 0.001). The forest map estimated by comprehensive effect size is shown in Figure 9. Subgroup analysis showed that there was no significant difference in anxiety scores between HADS subgroup (*SMD* = −0.31, 95%CI: −0.94–0.32, *P* = 0.337), and there was high heterogeneity between HADS subgroup (*I*^2^ = 78.0%, *P* = 0.003). Meta-regression analysis of HADS subgroup excluded the influence of publication years (*P* = 0.627). The results of sensitivity analysis were shown in Figure 10. When studies that had the greatest impact on heterogeneity were excluded [Xiu et al. (32) used the Chinese-Cantonese version of HADS scale], heterogeneity was reduced to negligible (*I*^2^ = 0.0%, *P* = 0.957). However, the difference between the two groups was still not statistically significant (*SMD* = −0.004, 95%CI: −0.394–0.386, *P* = 0.985), and the forest map of effect size estimation was shown in Figure 11. Egger's test results are shown in Figure 12, without significant publication bias (*P* = 0.339).

## Discussion

### Clinical implications

This study applied a systematic and rigorous search strategy to retrieve relevant studies according to the research objectives. A meta-analysis was conducted on QOL, depression, or anxiety of informal cancer caregivers treated with CBT. The RCTs included were not well-controlled in terms of blinding and allocation concealment methods. Given the nature of the clinical environment and possible ethical issues in these studies, blind allocation of participants and key personnel was almost impossible. However, it was assumed that as long as the effects of the intervention were not interfered by intergroup contamination, the results were less likely to be affected by the lack of blinding.

Three outcome indicators were extracted: QOL, depression, and anxiety. Subgroup analysis, meta-regression, and sensitivity analysis were performed to eliminate the bias caused by confounders for QOL, but the results were not satisfactory. After excluding the RCTs that had a large impact on heterogeneity, the difference between the two groups was still not statistically significant. However, the result was stably reliable, that is, from the statistical sense, the improvement effect of CBT on the QOL of the informal cancer caregivers was not obvious. Regarding the effects of CBT on depression, the combined effect size showed significant differences between the two groups, but the results showed moderate heterogeneity. Subgroup analysis showed that the HADS subgroup had significant differences (*P* = 0.002), but with moderate heterogeneity, while the CES-D subgroup showed no significant differences in depression scores between the two groups, with no heterogeneity. Therefore, we suggested that the effect of CBT on depression in informal cancer caregivers might be influenced by different measurement tools, in addition to the specific intervention content of CBT. Carefully selecting measurement tools, such as HADS, to assess depression may be more meaningful in the future. The overall effect of CBT on anxiety was statistically different, but the difference was no longer significant after the literature was excluded by subgroup and sensitivity analyzes. These were not consistent with the results of previous studies. One study (34) involved the meta-analysis of eight RCTs. The results showed that CBT improved the QOL (*P* = 0.008), but the result was limited to the application of CQOL-C to assess the integration of the three studies. Depression were discussed by descriptive analysis. This study included more RCTs and extracted more outcome indicators, and CBT interventions used in RCTs were different. Therefore, this study from clinical sense could not completely deny the effect of CBT intervention measures.

By summarizing the included RCTs, we found that not all CBT interventions were effective in terms of QOL, depression, or anxiety among informal cancer caregivers (19, 30), and these interventions that were effective were not all consistently maintained during follow-up (32). Three RCTs evaluated the sleep status of the participants and showed good results (21, 32), but were not included in the meta-analysis due to the limited number. In addition, the CBT intervention aimed at couples were found effective and remained stable during 3 months (17, 18) and 4 months (22) of follow-up. RCTs for informal caregivers of patients with cancer in palliative care found that CBT interventions were promising and the earlier they were introduced, the better, especially for caregivers with severe emotional problems (27). In addition, some interventions were conducted via the Internet or mobile phones and all had positive effects on the participants, despite a lack of comparison with face-to-face interventions, which was significant in the rapidly developing era of Internet communication (22, 27, 33).

### Study limitations

This study had some non-negligible limitations. The most important was the limited number of studies included. We only searched the published Chinese and English literature, excluding gray literature, non-indexed journals, or studies in languages other than Chinese and English, leading to reporting bias. In addition, the included RCT intervention plans, intervention time, measurement tools, outcome indicators, and so on were inconsistent. Therefore, it was difficult to compare the results between studies and the overall level of examination evidence, resulting in bias in the final results. Second, except for the CES-D subgroup of depression, the combined effect sizes of other outcome indicators were highly heterogeneous. Excluding the influence of publishing years and time span, the heterogeneity might be caused by the different research objects (cancer types, stages, and age were not the same), different CBT intervention contents (theoretical basis, implementation methods, intervention time, and so on were not the same), same ending index selection of different measurement tools, and many other factors. Third, this study did not account for the fact that different CBT intervention might bring about different effects on informal cancer caregivers. For example, the CBT interventions by face to face and mobile phones might be problematic in evaluating efficacy. Fourth, some studies did not carry out follow-up evaluation and the follow-up time and evaluation indexes were not consistent in the follow-up studies; hence, the overall long-term intervention effect could not be counted. Hence, it is difficult to provide specific recommendations for certain timings and frequency of the interventions.

### Conclusions

This meta-analysis showed that CBT had a positive effect in terms of reducing depression and anxiety in informal cancer caregivers, did not reach statistical significance for QOL, but showed positive trends. The literature included in this study was limited, and hence its conclusions need to be further confirmed by more large-sample, multicenter randomized controlled studies. In the future, studies should be conducted according to the specific situation of the research object, including the type of cancer, cancer stage, relationship between patients and caregivers, sex of the caregiver, and so on. They should generate corresponding intervention content, have an appropriate evaluation time, and select reasonable measurement tools to enhance the QOL of informal cancer caregivers.

## Author contributions

YW and YB conceptualized the research. SZ and QW conducted the literature search. SZ and GY were responsible for data extraction and carried out the meta-analysis. SZ drafted the protocol and the initial manuscript while HR reviewed them and made suggestions for revisions. All authors revised and approved the final version.

## Funding

This study was funded by National Science and Technology Support Program (Grant No. 2021ZD0200701) and Project of Hebei Provincial Finance Department (LNB202016).

## Conflict of interest

The authors declare that the research was conducted in the absence of any commercial or financial relationships that could be construed as a potential conflict of interest.

## Publisher's note

All claims expressed in this article are solely those of the authors and do not necessarily represent those of their affiliated organizations, or those of the publisher, the editors and the reviewers. Any product that may be evaluated in this article, or claim that may be made by its manufacturer, is not guaranteed or endorsed by the publisher.

## Appendix

Appendix 1 Brief introduction of the scale. CQOL-C: The scale is a 35-item, 5-point Likert-type scale. A higher score indicates a better quality of life. CQOLC has proven to be an effective tool for measuring the quality of life of cancer caregivers, with a Cronbach α coefficient of 0.91. COH-QOL: The 41-item scale measures overall QOL and four subareas: physical, mental, social and spiritual health. The Family Edition is a 37-item sequential tool for measuring the quality of life of family members caring for cancer patients. WHOQOL: The WHOQOL is an international scale developed by the World Health Organization to measure the health-related quality of life of individuals. The scale not only has good psychometric properties such as reliability, validity and responsiveness, but also has international comparability, that is, the quality of life scores measured in different cultural backgrounds are comparable. POMS: The 65-item scale measures six emotional dimensions: tension, anger, depression, vitality, fatigue and confusion; It has been widely used in clinical oncology research. The internal consistency coefficient of the scale ranged from 0.77 to 0.87, and the 3-week retest coefficient of cancer patients ranged from 0.62 to 0.80. CES-D: The Chinese version of CES-D is suitable for different age groups in China, and it is a reliable and effective self-rated depression symptom measurement tool. The Cronbach α coefficient was 0.90; the Cronbach α coefficient for each factor was 0.68–0.86. BSI: The scale contains 53 items, covering nine symptom dimensions: somatization, compulsion, interpersonal sensitivity, depression, anxiety, hostility, fear and anxiety, paranoia and psychoticism; and three overall depression indexes: the overall severity index, the positive symptom depression index, and the total positive symptom index. DASS-21: Dass-21 has the same stable factor structure and the same good reliability and validity as the full version of DASS, and the reliability test results of DASS-21 are satisfactory. The internal consistency of the three subscales ranged from 0.76 to 0.79, and the internal consistency of the total scale reached 0.89.It is more suitable as a tool for rapid screening in scientific research and clinic. HAMD: It is the most widely used scale in the assessment of depression, with a total of 17 assessment items. A 5-level score of 0–4 is adopted. HAMD >8 is classified as depression, and the higher the score, the more severe the depressive symptoms. HADS: The 14-item scale, which has been widely used as a screening tool for anxiety and depression in patients with advanced cancer, has been used in caregivers of cancer patients, and there is currently no uniform and accepted cut-off point.
